# Indicators of psychomotor development of premature infants by perinatal CNS lesion

**DOI:** 10.1192/j.eurpsy.2021.2006

**Published:** 2021-08-13

**Authors:** G. Abdullayeva, S. Batyrkhanov, M. Abdullaeva

**Affiliations:** 1 Department Of Propaedeutic Of Childhood Diseases, Kazakh National Medical University after Asfendiyarov, Almaty, Kazakhstan; 2 Psychology, Lomonosov Moscow State University, Moscow, Russian Federation

**Keywords:** premature infants, hypoxic-ischemic damage, psychomotor development

## Abstract

**Introduction:**

The birth premature babies with hypoxic-ischemic damage to the neutral system with the subsequent development of hypoxic encephalopathy (HIE). Monitoring of the mental development and neurological status of such prematurely born children is carried out taking into account the corrected age and traditional scales.

**Objectives:**

To compare indicators of psychomotor development in preterm infants (gestational age < 32 weeks) with and without hypoxic-ischemic encephalopathy.

**Methods:**

A prospective study was carried out in the neurological department. The study included data from infants with a gestational age of < 32 weeks of gestation. Scale score immediately after birth and at corrected ages in the first, third and sixth months of life (data analysis according to Griffiths Scales).

**Results:**

Data from 95 newborns were eligible for conclusion. Of these, 67 children took part in the study, 32,8% of them were diagnosed with hypoxic-ischemic encephalopathy. In newborns with HIE gestational age at birth was less so they received parenteral nutrition for a longer time, the body weight gained during the hospital stay was less, they needed more time to switch to enteral nutrition. And only at the 3^rd^ (80% of children) and 6^th^ months of life, there were no statistically significant differences in psychomotor development between groups with and without hypoxic-ischemic encephalopathy.

**Conclusions:**

In this study, it was shown that in premature infants with hypoxic-ischemic encephalopathy, normal indicators of psychomotor development and neurological status were restored at the corrected age only by 6 months of age.

**Disclosure:**

No significant relationships.

